# Validation of machine learning angiography-derived physiological pattern of coronary artery disease

**DOI:** 10.1093/ehjdh/ztaf031

**Published:** 2025-04-08

**Authors:** Yueyun Zhu, Simone Fezzi, Norma Bargary, Daixin Ding, Roberto Scarsini, Mattia Lunardi, Antonio Maria Leone, Concetta Mammone, Max Wagener, Angela McInerney, Gabor Toth, Gabriele Pesarini, David Connolly, Carlo Trani, Shengxian Tu, Flavio Ribichini, Francesco Burzotta, William Wijns, Andrew J Simpkin

**Affiliations:** School of Mathematical and Statistical Sciences, University of Galway, University Road, Galway H91 TK33, Ireland; The Lambe Institute for Translational Medicine, The Smart Sensors Laboratory and Curam, University of Galway, University Road, Galway H91 TK33, Ireland; Division of Cardiology, Department of Medicine, University of Verona, Verona, Italy; Department of Mathematics and Statistics, University of Limerick, Castletroy, Limerick V94 T9PX, Ireland; The Lambe Institute for Translational Medicine, The Smart Sensors Laboratory and Curam, University of Galway, University Road, Galway H91 TK33, Ireland; Division of Cardiology, Department of Medicine, University of Verona, Verona, Italy; Department of Mathematics and Statistics, University of Limerick, Castletroy, Limerick V94 T9PX, Ireland; Department of Cardiovascular Sciences, Fondazione Policlinico Universitario A. Gemelli IRCCS, Università Cattolica del Sacro Cuore, Rome, Italy; Center of Excellence in Cardiovascular Sciences, Ospedale Isola Tiberina, Gemelli Isola, Università Cattolica del Sacro Cuore, Roma, Italy; Department of Cardiology, University Heart Center Graz, Medical University Graz, Graz, Austria; School of Biomedical Engineering, Biomedical Instrument Institute, Shanghai Jiao Tong University, Shanghai, China; Division of Cardiology, Department of Medicine, University of Verona, Verona, Italy; Department of Cardiovascular Sciences, Fondazione Policlinico Universitario A. Gemelli IRCCS, Università Cattolica del Sacro Cuore, Rome, Italy; Center of Excellence in Cardiovascular Sciences, Ospedale Isola Tiberina, Gemelli Isola, Università Cattolica del Sacro Cuore, Roma, Italy; Division of Cardiology, Department of Medicine, University of Verona, Verona, Italy; School of Mathematical and Statistical Sciences, University of Galway, University Road, Galway H91 TK33, Ireland; School of Mathematical and Statistical Sciences, University of Galway, University Road, Galway H91 TK33, Ireland; Department of Cardiology, University Heart Center Graz, Medical University Graz, Graz, Austria; Division of Cardiology, Department of Medicine, University of Verona, Verona, Italy; The Lambe Institute for Translational Medicine, The Smart Sensors Laboratory and Curam, University of Galway, University Road, Galway H91 TK33, Ireland; Department of Cardiovascular Sciences, Fondazione Policlinico Universitario A. Gemelli IRCCS, Università Cattolica del Sacro Cuore, Rome, Italy; School of Biomedical Engineering, Biomedical Instrument Institute, Shanghai Jiao Tong University, Shanghai, China; Division of Cardiology, Department of Medicine, University of Verona, Verona, Italy; Department of Cardiovascular Sciences, Fondazione Policlinico Universitario A. Gemelli IRCCS, Università Cattolica del Sacro Cuore, Rome, Italy; The Lambe Institute for Translational Medicine, The Smart Sensors Laboratory and Curam, University of Galway, University Road, Galway H91 TK33, Ireland; School of Mathematical and Statistical Sciences, University of Galway, University Road, Galway H91 TK33, Ireland

**Keywords:** Coronary physiology, Machine learning models, Physiological pattern of disease, Percutaneous coronary intervention

## Abstract

**Aims:**

The classification of physiological patterns of coronary artery disease (CAD) is crucial for clinical decision-making, significantly affecting the planning and success of percutaneous coronary interventions (PCIs). This study aimed to develop a novel index to reliably interpret and classify physiological CAD patterns based on virtual pullbacks from single-view Murray’s law-based quantitative flow ratio (μFR) analysis.

**Methods and results:**

The pullback pressure gradient index (PPGi) was used to classify CAD patterns, with a cut-off value of PPGi = 0.78 distinguishing focal from diffuse and non-focal disease. The machine learning methods using penalized logistic regression and random forest were proposed to assess CAD patterns. Scores derived from multivariate functional principal component analysis of μFR and quantitative coronary analysis improved model performance. Expert panel interpretations served as the reference. A total of 343 vessels (291 patients) underwent classification. The PPGi cut-off of 0.78 achieved 67% accuracy [95% confidence interval (CI): 66–68%] for focal vs. diffuse and 76% accuracy (95% CI: 75–76%) for focal vs. non-focal classification. The penalized logistic regression model, including PPGi as a feature, provided superior accuracy: 88% (95% CI: 87–88%) for focal vs. diffuse and 81% (95% CI: 80–81%) for focal vs. non-focal classification. Moreover, the random forest model with PPGi as one of the features was applied for multiclass classification, providing an accuracy of 73% (95% CI: 73–73%).

**Conclusion:**

The machine learning models for physiological patterns of CAD classification outperformed the binary PPGi method, providing robust and generalizable classification across different study populations.

## Introduction

The classification of physiological patterns of coronary artery disease (CAD), including focal, serial lesions, diffuse disease, and mixed patterns, has become increasingly important in clinical decision-making.^1–5^ Recent studies have investigated the role of coronary physiological indices in assessing CAD patterns, such as wire-based fractional flow reserve (FFR), instantaneous wave-free ratio (iFR), or quantitative flow ratio (QFR) computed from the coronary angiogram.^1–5^ Different mathematical metrics have been used to classify physiological patterns, including FFR gradient per unit time (dFFR[t]/dt) and pullback pressure gradient index (PPGi).^4,6–11^ These metrics were applied to determine the disease severity along the course of the vessel and predict post-PCI outcomes. Both these metrics are calculated based on pressure-wire (PW)-pullback performed during continuous hyperaemia. Pullback pressure gradient index quantitatively measures the physiological distribution of coronary plaques along the vessel and is capable of distinguishing between focal and diffuse disease, while dFFR[t]/dt reflects the local physiological disease severity. Despite the extensive evidence in support of physiological assessment as a gatekeeper for coronary revascularization, and the growing one in support of physiology guidance for percutaneous coronary intervention (PCI) optimization, its use remains hampered by economic and logistic reasons (i.e. need for a PW and for hyperaemic agents, increased costs, and procedural time).^12–14^ Notably, both the PPGi and dFFR[t]/dt metrics require automated motorized PW-pullback, further limiting their applicability in real-world practice. To overcome such limitations, angiography-derived physiological indices, such as the Murray’s law-based quantitative flow ratio (μFR), have been recently developed and validated.^3,15^ Murray’s law-based quantitative flow ratio provides an inherently co-localized virtual pullback that qualitatively interprets the physiological pattern of disease (focal vs. diffuse vs. mixed vs. serial).^2,3,16,17^ A lack of consensus in the qualitative interpretation of physiological pattern definition is evident, making it challenging to reach a standardization, relying on operator interpretation and experience.

The aim of this study was to propose a novel machine learning method to quantify and classify physiological patterns, which can be reproducible and generalized across a range of study samples, having qualitative interpretation as a reference standard.

## Methods

### Study population

This is a proof-of-concept, validation study including a patient-level pooled analysis of two prospective cohorts of patients with de-novo CAD that underwent clinically driven PCI, enrolled at two European centres [Verona University Hospital, Verona, Italy (185 CESC); Policlinico Gemelli, Rome, Italy (FORZA NCT01824030)] between December 2012 and September 2020. Coronary angiograms were anonymized, transferred, and analysed in the independent Smart-Sensors laboratory (The Lambe Institute of Translational Research, University of Galway, Ireland) in a blinded fashion, by experienced and certified analysers. Patients younger than 18 years old, with known contra-indication to dual anti-platelet therapy, a concomitant indication to open-heart surgery, heart-transplanted patients with allograft vasculopathy, or patients presenting with resuscitated cardiac arrest and women with childbearing potential were excluded from the study.

The study was conducted in accordance with the ethical principles of the Declaration of Helsinki, and it was approved by the institutional ethical board of the enrolling centres. All the patients had provided their written consent for the anonymous data collection.

Overall, the study utilized coronary angiographic data from 291 patients (343 vessels) included in previously reported studies.^18^ For each patient, information such as age, gender, smoking status, hypertension status, and diabetes status was provided.

### Murray law-based quantitative flow ratio analysis

Coronary angiograms were anonymized, sent, and centrally analyzed. Computation of μFR was performed using the μFR software (AngioPlus Core, version V3, Pulse Medical, Shanghai, China) by an experienced analyst, blinded to all clinical data. Angiographic exclusion criteria were ostial disease in the left main or in the right coronary artery, target vessel with collateral circulation or coronary flow from patent surgical grafts, target vessel with myocardial bridging, target vessel with previous myocardial infarction, and poor angiography image quality, including much overlap, foreshortening, incomplete contrast filling, or blurry lumen contours. The detailed methodology for single-view μFR computation has been described previously.^19^ A standard cut-off value of μFR (≤0.80) was used to classify the haemodynamic significance. For each vessel, fundamental descriptors such as lesion length, diameter, and %DS at the quantitative coronary analysis (QCA) were included.

### Physiological pattern of coronary artery disease interpretation

Physiological patterns of CAD indexes were derived from μFR virtual pullback traces: physiological distribution was assessed through the μFR virtual pullback PPGi which discriminates predominantly focal from predominantly diffuse disease, providing a continuous metric based on the magnitude of maximum pressure drop over 20 mm and on the extent of functional disease over the entire interrogated vessel.

Pullback pressure gradient index was calculated as follows:


$$\frac{\left[\frac{\mathrm{MaxPPG}i_{20\mathrm{mm}}}{\Delta \mu FR_{\mathrm{vessel}}}+\left(1-\frac{\mathrm{Lenght}\;\mathrm{with}\;\mathrm{functional}\;\mathrm{diseas}e_{\left(\mathrm{mm}\right)}}{\mathrm{Total}\;\mathrm{vessel}\;\mathrm{lengt}h_{\left(\mathrm{mm}\right)}}\right)\right]}{2}$$

As previously reported,^16^ high PPGi values (close to 1) suggest predominantly focal disease, whereas low values (close to 0) predominantly diffuse disease. A PPGi cut-off value (0.78) was used to dichotomize CAD into focal (PPGi ≥ 0.78) and diffuse (PPGi <0.78) disease, as previously validated.^6,20^

Local physiological severity was calculated by the instantaneous μFR gradient per unit length (dμFR/ds), using a cut-off value of 0.025/mm to identify the presence (≥0.025/mm) or the absence (<0.025/mm) of major gradients, as previously validated.^6,20^

In order to develop a novel machine learning-based computational tool for the interpretation of physiological pattern of CAD, μFR values were extracted from the pressure tracing every 0.35 mm. Together with the μFR values along the vessels’ length, a point-by-point (every 0.35 mm) reconstruction of further QCA-derived variables was obtained (i.e. minimal lumen diameter, reference vessel diameter). Vessels with distal μFR > 0.95 were considered without functional disease and excluded from the current analysis.

### Qualitative physiological patterns interpretation

**Figure 1:**
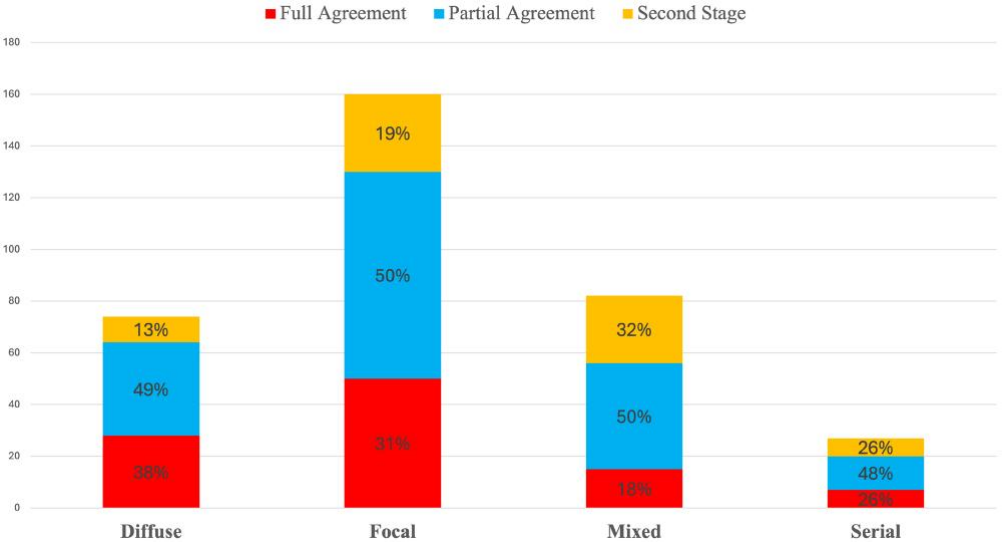
Qualitative interpretation of coronary artery disease patterns obtained from eight experienced interventional cardiologists. At the first round assessment, full agreement was considered a consensus reached by all eight cardiologists, but partial as the agreement from at least five cardiologists. Vessels with <5/8 concordant interpretations underwent a second stage of assessment until the divergences between the cardiologists were solved. The determinations made by the eight expert cardiologists were regarded as the reference standard, which was utilized to train the machine learning algorithm and provide the classification results obtained in the later sections.

In this study, 343 vessels were assessed independently by eight expert cardiologists, with significant experience in acquisition and interpretation of coronary physiology. Using a dedicated offline portal, experts interpreted each trace independently, without access to the coronary angiogram or additional clinical information, resulting in a qualitative interpretation of physiological pressure drop over the vessel, using previously proposed definitions as a reference.^17^ Each expert labelled each vessel as having one of four distinct physiological CAD patterns, namely focal lesion, diffuse disease, mixed pattern, and serial lesions. The final decision for each physiological pattern was determined by the interpretation provided from the eight cardiologists, defined as the agreement of at least five out of eight (see *Figure 1*). At the first round assessment, there were 100 vessels (29%) with a consensus reached by all eight cardiologists and 170 vessels (50%) with an agreement from at least five cardiologists. The remaining 73 vessels (21%) with <5/8 concordant interpretations underwent a second stage of assessment until the divergences between the cardiologists were solved. We believe using a single expert would lead to issues of low reproduciblity. Alternatively, using PPGi cut-off value 0.78 would lead to a suboptimal ground truth. Consequently, the determinations made by the eight expert cardiologists were regarded as the reference standard, which was utilized to train the machine learning algorithm and provide the classification results obtained in later sections.^21^

### Study endpoints

Overall, the study aimed to develop a machine learning method for physiological pattern interpretation based on a single-view angiography-derived physiological assessment, in order to attain high-level classification performance and reproducibility.

The first physiological pattern of CAD classification concentrated on differentiating focal from diffuse disease. The second classification dealt with focal vs. non-focal patterns (combining diffuse, mixed, and serial lesions). Additionally, a multiclass classification was performed with three categories: focal, diffuse, and others (combining mixed and serial lesions). The sample size for serial lesions was limited, accounting for only 8% (see *Table 1*). It seemed more reasonable to combine serial and mixed patterns into one category. The multiclass classification was therefore refined to distinguish among focal, diffuse, and others.

**Table 1:** Baseline characteristics

	Total (*n* = 343)	Diffuse 74 (22%)	Focal 160 (47%)	Mixed 82 (24%)	Serial 27 (7%)
Patient characteristics					
Age	62 ± 13	68 ± 11	58 ± 13	64 ± 12	61 ± 12
Sex male	283 (83%)	53 (19%)	144 (51%)	61 (22%)	25 (8%)
Non smoker	153 (45%)	42 (28%)	66 (43%)	34 (22%)	11 (7%)
Current smoker	154 (45%)	31 (20%)	71 (46%)	40 (26%)	12 (8%)
Previous smoker	36 (10%)	1 (3%)	23 (64%)	8 (22%)	4 (11%)
Hypertension	238 (69%)	60 (25%)	95 (40%)	60 (25%)	23 (10%)
Diabetes	73 (21%)	18 (25%)	25 (34%)	27 (37%)	3 (4%)
Dyslipidaemia	237 (69%)	53 (22%)	103 (43%)	62 (26%)	19 (9%)
Clinical presentation					
Chronic coronary syndrome	160 (47%)	52 (33%)	60 (37%)	37 (23%)	11 (7%)
Unstable angina	18 (5%)	1 (5%)	13 (73%)	2 (11%)	2 (11%)
Myocardial infarction	165 (48%)	21 (13%)	87 (53%)	43 (26%)	14 (8%)
Vessel characteristics					
μFR	0.74 ± 0.14	0.82 ± 0.08	0.71 ± 0.16	0.74 ± 0.11	0.68 ± 0.15
dμFR/ds	0.05 ± 0.05	0.02 ± 0.01	0.08 ± 0.06	0.05 ± 0.04	0.06 ± 0.04
PPGi	0.69 ± 0.13	0.59 ± 0.11	0.78 ± 0.09	0.62 ± 0.09	0.65 ± 0.09
Diameter	3.74 ± 0.74	3.57 ± 0.69	3.75 ± 0.78	3.84 ± 0.69	3.79 ± 0.69
Reference diameter	3.61 ± 0.66	3.65 ± 0.63	3.56 ± 0.68	3.64 ± 0.6	3.76 ± 0.84

Values are mean ± standard deviation or *n* (%).

dμFR/ds, instantaneous μFR gradient per unit length; μFR, Murray’s law-based quantitative flow ratio; PPGi, pullback pressure gradient index.

## Statistical analysis

In this study, two penalized logistic models with elastic net regularization^22^ were developed, (see Supplementary material online, *Table S1*). The first model, namely μFR model, included 12 clinical and procedural features. Six features were related to demographical information (age, gender, smoking, diabetes, dyslipidaemia, and hypertension status), three features were the scores estimated from multivariate functional principal component analysis (MFPCA), one feature was the reference diameter, and the last two features were μFR and dμFR/ds. The second model, named PPGi model, extended μFR model by including an additionally feature PPGi. For comparison, random forest, a commonly used machine learning algorithm, was also applied to classify CAD patterns. Detailed statistical methods and models are described in Supplementary Materials.

All analyses were performed in R version 4.3.0 (R Foundation for Statistical Computing). The package MFPCA^23^ was used to calculate multivariate functional principal components and scores. The package glmnet^24^ was used for the penalized logistic regression model. The overall performance of the models on classification was assessed by accuracy, sensitivity, specificity, positive predictive value (PPV), negative predictive value (NPV), and area under the curve (AUC) values.

## Results

### Baseline characteristics

The baseline characteristics of the study population (291 patients with 343 vessels) were summarized in *Table 1*. The mean age was 62 ± 13 years, and 83% of the patients were men. Based on the experts committee’s adjudication, 160 vessels (47%) were deemed to have focal CAD pattern and 183 (53%) were identified with non-focal patterns. Within the non-focal patterns, 74 vessels were classified as diffuse. Therefore, a total of 234 vessels were assessed to have either focal or diffuse CAD patterns, with an approximate 2:1 ratio of focal to diffuse.

For focal and serial lesions, μFR values were lower than the average for the entire population. A PPGi value was significantly lower in diffuse patterns, as compared to focal lesions (0.50 ± 0.11 vs. 0.78 ± 0.09; *P*-value <0.01).

### Binary classification using pullback pressure gradient index cut-off values

**Figure 2:**
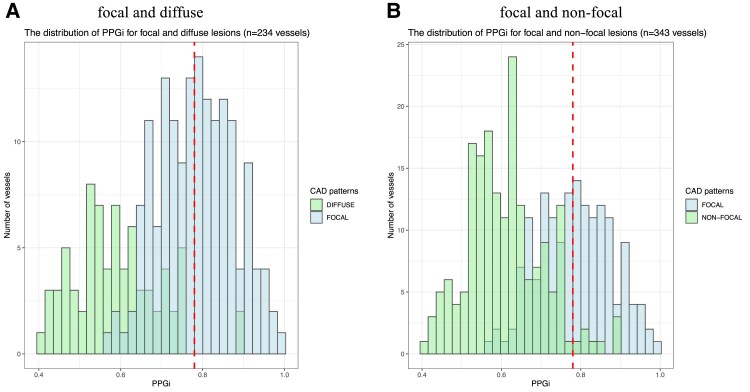
Distribution of pullback pressure gradient index and its cut-off value. Part A shows the distribution of pullback pressure gradient index values for vessels with focal and diffuse disease. Part B shows the distribution of pullback pressure gradient index values for vessels with focal and non-focal disease. The red dashed lines represent the value of pullback pressure gradient index = 0.78. CAD, coronary artery disease; PPGi, pullback pressure gradient index.

Based on the decisions from eight cardiologists, 234 vessels were adjudicated to have focal or diffuse patterns. The distribution of PPGi across these 234 vessels is shown in *Figure 2A*, which indicates the distribution for diffuse has a longer tail, whereas the distribution for focal appears approximately symmetric. The red dashed line in *Figure 2* is the cut-off value of PPGi = 0.78. Following this, the 234 vessels were subjected to the classification, of which 84 focal (sensitivity 53%) and 72 diffuse (specificity 97%) were correctly classified, leading to an accuracy of 67% [95% confidence interval (CI): 60–73%], with PPV at 98% and NPV at 49%.

The distribution of PPGi across 343 vessels with focal and non-focal patterns is shown in *Figure 2B*, with the red dashed line at value of 0.78. By using PPGi = 0.78 as the cut-off to distinguish focal vs. non-focal disease, 84 focal (sensitivity 53%) and 175 non-focal (specificity 96%) were correctly classified, resulting in an accuracy of 76% (95% CI: 71–80%), with PPV at 91% and NPV at 70%.

### Binary classification using Murray’s law-based quantitative flow ratio model

**Figure 3:**
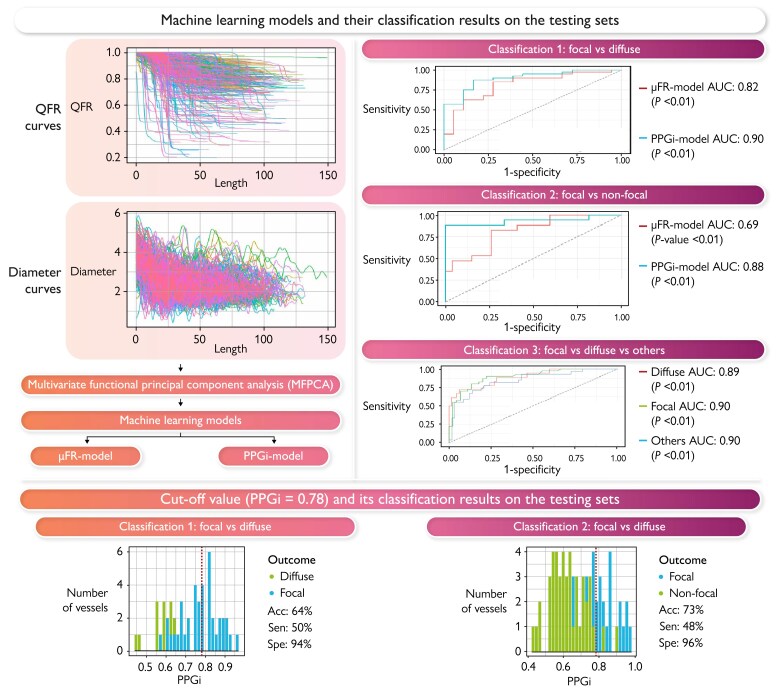
The classification results based on machine learning models and the cut-off pullback pressure gradient index value. The top panel illustrates the classification results from two machine learning models. Multivariate functional principal component analysis is applied to Murray’s law-based quantitative flow ratio and quantitative coronary analysis diameter curves to derive scores, which are subsequently used as features in the two models. For binary classifications, the receiver operating characteristic area under the curve indicates the pullback pressure gradient index model outperforms di. The area under the curve for multiclass from pullback pressure gradient index model is presented in the third classification. The bottom panel shows the classification results by using cut-off value (pullback pressure gradient index = 0.78, the red dashed lines). For classification 1, all bars to the left (right) of 0.78 are classified as diffuse (focal). For classification 2, all bars to the left (right) of 0.78 are classified as non-focal (focal). AUC, area under the curve; μFR, Murray’s law-based quantitative flow ratio; PPGi, pullback pressure gradient index; MFPCA, multivariate functional principal component analysis; QCA, quantitative coronary analysis; Acc, accuracy; Sen, sensitivity; Spe, specificity.

**Figure 4:**
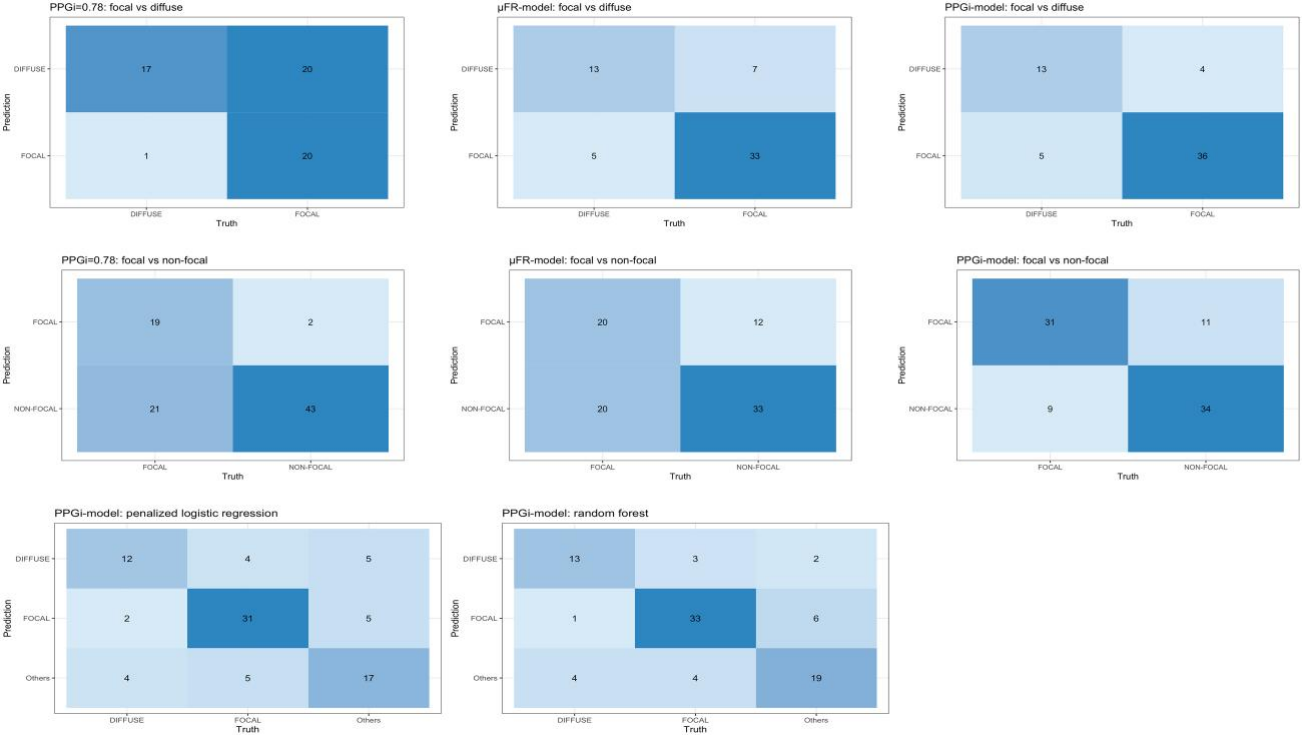
Confusion matrices on the testing sets using the three methods. The top, middle, and bottom panels illustrate the confusion matrices for focal vs. diffuse, focal vs. non-focal, and focal vs. diffuse vs. others, respectively. Note that pullback pressure gradient index = 0.78 represents the method using 0.78 as the cut-off value. For binary classification, the results from Murray’s law-based quantitative flow ratio model and pullback pressure gradient index model are based on penalized logistic regression algorithm. For multiclass classification, the results from pullback pressure gradient index model based on penalized logistic and random forest are provided. μFR, Murray’s law-based quantitative flow ratio; PPGi, pullback pressure gradient index.

To prevent model from overfitting, 234 vessels with focal and diffuse disease were randomly split into training (*n* = 176, 75%) and testing (*n* = 58, 25%) sets. The μFR model was fitted to the training data and used to classify the focal and diffuse patterns for the testing set. The μFR model based on penalized logistic regression achieved 79% accuracy (95% CI: 67–89%), with 33 focal (sensitivity 83%) and 13 diffuse (specificity 72%) correctly classified (see the top panel of *Figure 4*). Positive and negative predictive values were 87 and 65%, respectively. The receiver operating characteristic (ROC) curve with an AUC of 0.82 (*P*-value < 0.01) is provided in *Figure 3*. Moreover, the same testing set was assessed by the cut-off value of PPGi = 0.78, which correctly classified all 17 diffuse (specificity 94%) and 20 focal (sensitivity 50%) (see *Figure 4*), leading to an accuracy of 64%, a PPV of 95%, and an NPV of 46%. As shown in *Figure 4*, it was evident that μFR model distinguishes more focal lesions than using PPGi = 0.78 as the cut-off. Additionally, the μFR model based on random forest was also employed, resulting in a lower accuracy of 76% (95% CI: 63–86%) compared with the model based on penalized logistic regression.

For the classification of focal and non-focal vessels, 343 vessels were randomly partitioned into training (*n* = 258, 75%) and testing (*n* = 85, 25%) sets. The middle panel of *Figure 4* illustrates less non-focal lesions (*n* = 33, specificity 73%) were correctly classified from the μFR model based on penalized logistic regression, compared with using PPGi = 0.78 as the cut-off value (*n* = 43, specificity 96%). Only one more focal lesion was correctly classified from the μFR model. Overall, the μFR model based on penalized logistic regression provided an accuracy of 62% (95% CI: 51–73%), which was lower than using PPGi = 0.78 as the cut-off (73%, 95% CI: 62–82%). It is worth mentioning that the μFR model based on random forest yielded a lower accuracy of 61% (95% CI: 50–72%).

### Binary classification using pullback pressure gradient index model

The PPGi model extended the μFR model by including PPGi as one of the features. In order to compare the classification results, the same testing sets were used.

When considering the classification of focal vs. diffuse, the PPGi model based on penalized logistic regression yielded the highest accuracy (85%, 95% CI: 73–93%), with 13 diffuse (specificity 72%) and 36 focal (sensitivity 90%) lesions correctly classified. Positive and negative predictive values were 88 and 76%, respectively. The ROC curve with an AUC of 90% (*P*-value < 0.01) is provided in *Figure 3*. Consequently, including PPGi as one of the features in penalized logistic regression significantly improved the classification outcomes. For comparison, the PPGi model based on random forest was also employed, leading to an accuracy of 84% (95% CI: 73–93%).

In terms of the classification on focal vs. non-focal, the PPGi model resulted in an accuracy of 76% (95% CI: 66–85%), with 31 focal (sensitivity 78%) and 34 non-focal (specificity 76%) correctly classified (see the middle panel of *Figure 4*). The PPGi model based on penalized logistic regression outperformed other two models when dichotomizing CAD into focal and non-focal disease, with a PPV of 74%, an NPV of 79%, and an AUC of 0.88. For comparison, the PPGi model based on random forest provided similar classification outcomes, with an accuracy of 76% (95% CI: 66% to 85%), a PPV of 74%, an NPV of 79%, and an AUC of 0.85.

For binary classification using PPGi model, the penalized logistic regression is preferable, because it provides more interpretable results than the random forest, such as the regularization paths of coefficients and the selection of tuning parameters (see Supplementary material online, *Figures S2* and *S3*).

### Multiclass classification using pullback pressure gradient index model

According to the aforementioned results, it was evident that the PPGi model demonstrated superior performance over the μFR model and the cut-off method. To further explore the clinical significance, the PPGi model was also applied to multiclass classification.

**Figure 5:**
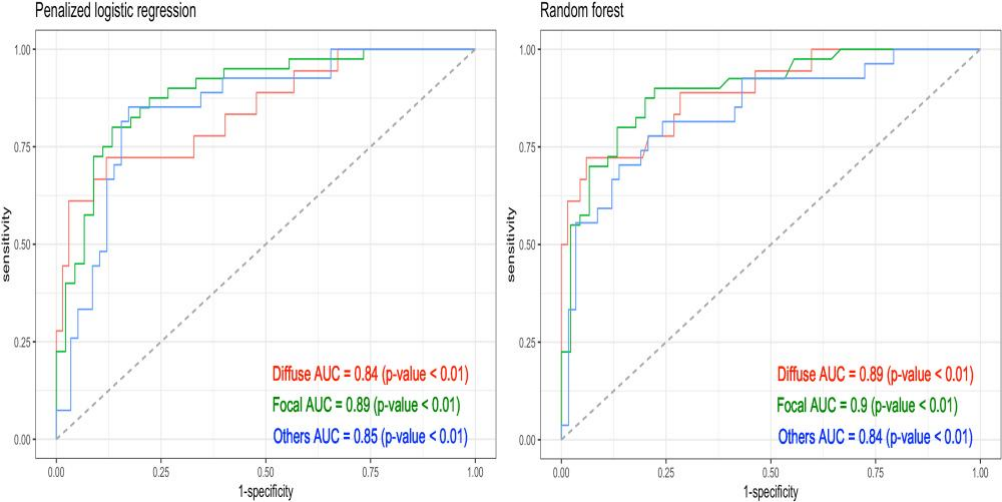
Receiver operating characteristic curves for diffuse vs. focal vs. others on the testing set using pullback pressure gradient index model. Left panel: pullback pressure gradient index model uses penalized logistic regression algorithm. Right panel: pullback pressure gradient index model uses random forest algorithm. ROC, receiver operating characteristic; AUC, area under the curve.

For the multiclass classification on focal, diffuse, and others (combining serial and mixed patterns), 343 vessels were randomly partitioned into training (*n* = 258, 75%) and testing (*n* = 85, 25%) sets. The bottom panel of *Figure 4* presents confusion matrices for the testing set, using PPGi model based on penalized logistic regression and random forest, respectively. It shows that random forest outperformed penalized logistic regression, with a higher accuracy 76% (95% CI: 66–85%), compared with 71% (95% CI: 60–80%). On the contrary, *Figure 5* presents the ROC curves obtained from penalized logistic regression and random forest, respectively, using a one-vs.-rest approach. The random forest provides higher AUC values than penalized logistic regression. Consequently, PPGi model based on random forest was the final model to perform multiclass classification.

### Classification results after 500 iterations

Repeatedly splitting the data into training and testing sets helps to assess the robustness and generalizability of the model. *Table 2* lists the classification performance of the three methods after randomly splitting 234 vessels for focal and diffuse disease 500 times. Note that using the cut-off value of PPGi failed to provide the probability estimates, and the AUC value was therefore not available. *Table 2* indicates that the PPGi model performed the best classification, with an overall accuracy of 88% (95% CI: 87–88%), a PPV of 90% (95% CI: 90–90%), and an NPV of 83% (95% CI: 82–84%).

**Table 2:** Classification performance on focal vs. diffuse (after 500 iterations)

	Accuracy	Sensitivity	Specificity	PPV	NPV	AUC
PPGi = 0.78	0.67	0.53	0.97	0.98	0.48	
	(0.66–0.68)	(0.52–0.53)	(0.97–0.98)	(0.98–0.98)	(0.48–0.49)	
μFR model	0.81	0.88	0.67	0.86	0.72	0.89
	(0.81–0.82)	(0.87–0.88)	(0.66–0.68)	(0.85–0.86)	(0.71–0.73)	(0.88–0.89)
PPGi model	0.88	0.93	0.76	0.90	0.83	0.94
	(0.87–0.88)	(0.92–0.83)	(0.75–0.77)	(0.90–0.91)	(0.82–0.84)	(0.94–0.95)

μFR, Murray’s law-based quantitative flow ratio; PPGi, pullback pressure gradient index; AUC, area under the curve; NPV, negative predictive value; PPV, positive predictive value.

μFR model and PPGi model are based on penalized logistic regression.

Similarly, *Table 3* shows the classification performance of the three methods for focal vs. non-focal cases after 500 iterations. In this case, the classification result derived from setting PPGi = 0.78 as the cut-off was better than μFR model, whereas PPGi model still outperformed the others with accuracy 81% (95% CI: 80–81%), PPV 84% (95% CI: 80–81%), and NPV 81% (95% CI: 81–81%).

**Table 3:** Classification performance on focal vs. non-focal (after 500 iterations)

	Accuracy	Sensitivity	Specificity	PPV	NPV	AUC
PPGi = 0.78	0.76	0.53	0.96	0.92	0.70	
	(0.75–0.76)	(0.52–0.53)	(0.96–0.96)	(0.92–0.92)	(0.69–0.70)	
μFR model	0.67	0.56	0.77	0.69	0.67	0.74
	(0.67–0.68)	(0.55–0.57)	(0.77–0.78)	(0.69–0.70)	(0.66–0.67)	(0.74–0.75)
PPGi model	0.81	0.78	0.83	0.81	0.81	0.89
	(0.80–0.81)	(0.77–0.78)	(0.83–0.84)	(0.80–0.81)	(0.81–0.81)	(0.89–0.90)

μFR, Murray’s law-based quantitative flow ratio; PPGi, pullback pressure gradient index; AUC, area under the curve; NPV, negative predictive value; PPV, positive predictive value.

μFR model and PPGi model are based on penalized logistic regression.

At last, the multiclass classification results including PPGi model based on penalized logistic regression and random forest after 500 iterations are provided in *Table 4*. The random forest outperformed penalized logistic regression, with an accuracy of 73% (95% CI: 73–73%). Moreover, the sensitivity, specificity, and AUC values for diffuse and focal classes obtained from the random forest were higher than those obtained from the penalized logistic regression.

**Table 4:** Classification performance on focal vs. diffuse vs. others (after 500 iterations)

PPGi model	Accuracy	Diffuse			Focal			Others		
		Sen	Spe	AUC	Sen	Spe	AUC	Sen	Spe	AUC
PLR	0.7	0.59	0.89	0.87	0.85	0.78	0.89	0.56	0.86	0.80
	(0.70–0.71)	(0.58–0.61)	(0.89–0.89)	(0.87–0.88)	(0.84–0.85)	(0.78–0.79)	(0.89–0.89)	(0.55–0.57)	(0.86–0.86)	(0.80–0.81)
RF	0.73	0.63	0.91	0.91	0.86	0.80	0.90	0.60	0.87	0.79
	(0.73–0.73)	(0.62–0.64)	(0.91–0.91)	(0.90–0.91)	(0.86–0.87)	(0.79–0.80)	(0.90–0.90)	(0.59–0.61)	(0.86–0.87)	(0.79–0.80)

PPGi, pullback pressure gradient index; Sen, sensitivity; Spe, specificity; AUC, area under the curve; PLR, penalized logistic regression; RF, random forest.

PPGi model is based on penalized logistic regression and random forest, respectively.

## Discussion

In this study, we aimed to develop novel models based on machine learning algorithms to classify CAD patterns and to preliminarily test their performance. This concept may translate into a precious tool to identify CAD patterns in a reproducible, generalizable, and potentially automated way.

The main findings can be summarized as follows:

1. Pullback pressure gradient index with its validated cut-off (0.78) is a quantitative metrics that provides suboptimal accuracy in classifying binary physiological pattern of CAD.
2. Two models, the μFR model and PPGi model, based on machine learning algorithms penalized logistic regression and random forest, respectively, were developed to assess CAD patterns. Without including PPGi, the μFR model demonstrated lower performance compared with PPGi.
3. For binary classification, including PPGi in the penalized logistic regression model significantly improved the classification performance, leading to the best result among the three methods. The PPGi model achieved an accuracy, a PPV, and an NPV of 88% (95% CI: 87–88%), 90% (95% CI: 90–90%), and 83% (95% CI: 82–84%) for the classification of focal vs. diffuse and 81% (95% CI: 80–81%), 81% (95% CI: 80–81%), and 81% (95% CI: 81–81%) for the classification of focal vs. non-focal patterns.
4. For multiclass classification, including PPGi in the random forest model provides the best classification outcomes, with an overall accuracy of 73% (95% CI: 73–73%) and AUC values for diffuse (0.91, 95% CI: 0.90–0.91), focal (0.90, 95% CI: 0.90–0.90), and others (0.79, 95% CI: 0.79–0.80).

Functional patterns of CAD provide crucial information for PCI procedural planning and appropriateness. Diffuse disease poses several challenges in patients undergoing PCI including long coronary segments requiring treatment, lack of adequate landing zone for stenting, and impairment of coronary flow related to the diffuse atherosclerotic burden along the vessel.^17,25,26^ Moreover, diffuse disease is associated with higher risk of suboptimal functional result and residual symptoms after PCI.^20,27,28^ Therefore, the determination of the distribution pattern of disease with the identification of focal lesions or segments of diffuse disease has become one of the cornerstones of decision-making for myocardial revascularization.

Traditionally, at the PW-pullback, focal disease is defined as an abrupt pressure drop (delta FFR ≥0.05 or delta iFR ≥0.03) within a short vessel segment (≤20 mm), while serial/tandem lesions are defined as two or more focal stenoses separated by a non-diseased vessel segment >20 mm. Of note, the observed distribution pattern often combines features of focal and diffuse disease patterns.^17,25,26^ However, the qualitative interpretation of CAD patterns is subject to inter- and intra-operator variability, being potentially influenced by the expertise of physicians.^29^ In our original investigation, a complete agreement was achieved in only one-third of vessels, while in nearly one-fifth of the cases <5/8 concordant interpretations were achieved. Such difficulties in standardizing CAD patterns definitions and classification, together with technical and economic issues related to the need for a PW and hyperaemic agent, hamper the wide-spread adoption of coronary physiology in real-world practice.^30^

To overcome some of these limitations and standardize physiological pattern interpretation, quantitative metrics have been developed and validated. Pullback pressure gradient index quantitatively measures the physiological distribution of coronary plaques along the vessel and is capable of distinguishing between focal and diffuse disease. Collet *et al*.,^4^ in their preliminary experience, demonstrate PPGi reclassifies patterns interpretation in more than 30% of the lesions compared with angiography and it may facilitate the interpretation of FFR pullback traces. Another quantitative metric is the instantaneous FFR gradient per unit of time (dFFR[t]/dt), which allows identification local disease severity in terms of major gradients that are associated with better PCI functional result.^4,6–11,17^ Both these metrics are calculated based on PW-pullback performed during continuous hyperaemia and require automatized motorized PW-pullback, further limiting the applicability in real-world practice.

Angiography-derived physiological indices such as the μFR have been recently developed and validated to overcome several limitations of PW-assessment and lack of adoption in the real-world practice.^3,14,15^ The μFR results in an inherently co-localized virtual pullback that allows to qualitatively interpret the physiological pattern of disease (focal vs. diffuse vs. mixed vs. serial).^2,3,16,17^

The assessment of patterns based on angiography-derived physiology can be performed qualitatively by visual interpretation of the virtual pullback^2^ or quantitatively with the μFR-PPGi. A cut-off value μFR-PPGi < 0.78 was validated and seen to interact with long-term adverse events following PCI.^20^

According to our analysis, PPGi derived from μFR virtual pullback provides suboptimal accuracy in interpreting physiological pattern of CAD, while our penalized logistic regression model was seen to provide improved classification performance, leading to the best result among the three methods, both when distinguishing between focal vs. diffuse and/or focal vs. non-focal disease. On the contrary, for multiclass classification, including PPGi to random forest model yielded the best classification results, whereas using PPGi = 0.78 as a cut-off was not applicable in this case. The machine learning models are conceived as a powerful tool allowing a simple, fast, and reliable interpretation of CAD patterns, aiming to enhance precision medicine in terms of accurate diagnosis, procedural planning, and tailored treatment. Importantly, the application of MFPCA presents a potentially novel approach for CAD classification. The scores estimated from MFPCA summarized the joint variation between QFR and vessels’ diameter and therefore were included to the machine learning models to enhance the overall performance. Notably, the models can be reproduced and generalized across a range of study samples. In contrast, generalizing the cut-off PPGi values to other study populations would be problematic, especially when they are derived from a specific study sample, such as the mean or median values.^6,7^

Angiography-derived computational technologies have been conceived and developed to overcome some of the limitation of pressure wire-based physiological indices, aiding the possibility of deriving a physiological assessment from the simple coronary angiography, with no need for an invasive pressure wire, hyperaemia induction, and of inherently co-register physiology and angiography. Nevertheless, the recent FAVOR III Europe–Japan trial^31,32^ found that QFR failed to demonstrate non-inferiority to FFR for guiding PCI, with higher major adverse events rates (6.7 vs. 4.2%; hazard ratio 1.63, 95% CI: 1.11–2.41) and increased revascularizations (+21%) and stent implantations (+27%). Quantitative flow ratio identified more significant lesions with lower median values than FFR (0.81 vs. 0.84), particularly in the circumflex artery. While these results highlight limitations, QFR remains superior to angiography alone and could encourage greater use of physiology-guided PCI.^33^ Murray’s law-based quantitative flow ratio, an advanced QFR version based on Murray’s law, overcomes limitations such as vessel tapering and offers high diagnostic accuracy with FFR (93%) and enhanced feasibility using single-view angiography.^15,19,34^ Studies such as AQVA II^35^ and QUITE RIGHT^36,37^ show its superiority over angiography-guided PCI, achieving better physiological outcomes. Murray’s law-based quantitative flow ratio holds promise for fostering physiological guidance in centres where wire-based FFR is underutilized due to technical, economic, and procedural barriers.

## Study limitations

Our study has limitations. The first limitation is the lack of external prospective validation of the model. To address this limitation, we encourage other groups to replicate our work and plan to evaluate our machine learning models on a larger population in future studies ourselves.

Secondly, the assessment of CAD patterns from an individual expert cardiologist is likely to be subjective. However, using an independent panel of eight experienced cardiologists, we found 270 vessels (79%) were labelled as having the same CAD pattern by a majority of experts (*Figure 1*). Furthermore, the remaining 73 vessels (21%) without majority agreement underwent a second stage assessment to ensure the final labelled pattern was agreed upon (see yellow bars in *Figure 1*). The lack of full agreement among the cardiologists indicates that the decisions for these vessels were partly influenced by individual perspective. However, this fact highlights the requirement for an objective machine learning (ML) approach such as that developed in this study.

Finally, the cut-off value of PPGi is not applicable for multiclass classification and may be sensitive to the changes in population data for binary classification. On the contrary, the machine learning models should be considered a robust tool for the assessment of CAD patterns, and further studies would be required to validate its robustness across different study populations.

## Conclusions

Qualitative interpretation of physiological patterns of CAD is hampered by significant inter-observer variability. Angiography-derived PPGi provides suboptimal accuracy in interpreting physiological pattern of CAD. Our study introduced a novel machine learning tool for CAD pattern classification using angiography-derived physiology. The penalized logistic regression models, particularly those incorporating PPGi, demonstrated superior performance in distinguishing focal from diffuse disease. This method offers a reliable, reproducible, and generalizable approach to enhance precision in myocardial revascularization, potentially addressing limitations in current qualitative assessments and improving patient outcomes.

## Clinical perspectives

This study proposes a novel machine learning-based method to classify physiological patterns of CAD using angiography-derived physiological indices, overcoming conventional metrics such as FFR and angiography-derived PPGi. Fractional flow reserve and PPGi assessments have limitations, such as requiring dedicated pressure wires, hyperaemic agents, and motorized pullback, which restrict their use in clinical practice. The study introduces a penalized logistic regression model that incorporates PPGi and other vessel characteristics to improve the classification of CAD patterns into focal, diffuse, and non-focal types. The model was shown to outperform traditional PPGi-based classification, offering higher accuracy, sensitivity, and specificity. This machine learning approach has the potential to enhance decision-making in PCI by providing a more reliable and reproducible method for CAD pattern interpretation, integrating advanced computational methods into cardiovascular diagnostics to improve the precision and efficacy of treatment planning and optimization.

## Supplementary Material

ztaf031_Supplementary_Data

## Data Availability

The data underlying this article are available in the article and in its online supplementary material.
