# Supplementary material for: Validation of machine learning angiography-derived physiological pattern of coronary artery disease
Source: Eur Heart J Digit Health. 2025 Apr 8;6(4):577–86. doi: 10.1093/ehjdh/ztaf031 (PMC12282386; doi:10.1093/ehjdh/ztaf031)
Supplement: ztaf031_Supplementary_Data [file ztaf031_supplementary_data.zip › DH-D-24-00201_Supplementary material.docx]

**Supplementary Materials**

*2. Methods*

*2.4. Statistical analysis*

*Multivariate functional principal component analysis*

As μFR and diameter values were collected based on a point-by-point functional relationship with the length of the vessel, multivariate functional principal component analysis (MFPCA) was applied to these multivariate functional data, which consisted of μFR and diameter curves. The Central Illustration suggested the μFR curves exhibited most significant variation at the end of the vessels, whereas the diameter curves experienced predominant variation at the beginning of the vessels. Two important outcomes, multivariate functional principal components (MFPCs) and their scores, were calculated from MFPCA. Specifically, each element of the MFPCs captured the primary patterns of variation in the μFR and diameter curves about their means, respectively. The scores for each vessel were numerical values, which reflected the joint variation between μFR and diameter curves. In other words, the key features of μFR and diameter curves for an individual vessel were summarized together by these scores. Moreover, we selected 40 vessels based on their MFPC1 scores, including 20 vessels with higher scores (red lines) and 20 vessels with lower scores (blue lines), see Supplemental Figure 1. The vessels with higher MFPC1 scores were likely to exhibit more significant decreases in μFR values and have narrower diameters compared to those with lower MFPC1 scores. Consequently, the scores were carried forward as features of CAD patterns in penalized logistic regression and random forest models.

*Machine learning models*

Penalized logistic regression and random forest models were fitted to perform three classifications, namely focal vs diffuse, focal vs non-focal and focal vs diffuse vs others. For penalized logistic regression, elastic net regularization was applied to impose a penalty to shrink coefficients of the less important features towards zero (i.e. performing feature selection), which helped to reduce the complexity of the model and to prevent the model from overfitting. Let $\lambda$ be the regularization parameter which controls the overall strength of the penalty, and $\alpha$ be mixing parameter which bridges the gap between lasso regression ($\alpha=1)$ and ridge regression ($\alpha=0)$. For each observation, $w_{i}$ and $l\left( y_{i},\beta_{0}+\beta^{T}x_{i} \right)$ represent the weight and negative log-likelihood. In theory, the elastic net solves the problem:

$$\min_{\beta_{0},\beta} \frac{1}{N}\sum_{i=1}^{N} w_{i}l\left( y_{i},\beta_{0}+\beta^{T}x_{i} \right)+\lambda\left[ \left( 1-\alpha\right)||\beta{||}_{2}^{2}/2+\alpha||\beta{||}_{1} \right].$$

In addition to penalized logistic regression, random forest was also applied in the study. Random forest is a popular machine learning algorithm that is used for prediction and classification. Random forest achieves high accuracy by combining the predictions of multiple decision trees. However, the main limitation is the lack of interpretability. Random forest is considered ‘black-box’ models because they do not provide the estimated coefficients of predictors, which makes it difficult to explain the relationships between predictors and the response.

On the other hand, penalized logistic regression models provide the estimated coefficients. The regularization paths for coefficients as well as the selection for tuning parameters of the μFR-model and PPGi-model are presented in Supplemental Figure 2 and Figure 3, respectively.

Supplemental Figure 1 Multivariate functional principal component analysis (MFPCA)

The first three MFPCs are provided in the top left corner, where the black lines represent the mean functions of diameter and QFR curves, and the red (blue) lines are the MFPCs added to (subtracted from) the mean functions. The boxplots for the corresponding scores for four CAD patterns are provided in the bottom left corner. 40 curves are randomly selected and plotted based on their first MFPC score, where the red (blue) lines represent the curve with higher (lower) score.

MFPCs, multivariate functional principal components; μFR, Murray’s law-based quantitative flow ratio

Supplemental Figure 2 Regularization paths for model coefficients

The top and bottom panels show the regularization paths for the two models used for classification on focal vs diffuse and focal vs non-focal, respectively. Each coloured line shows the regularization path of one feature in the model as the regularization parameter varies.

μFR, Murray’s law-based quantitative flow ratio; PPGi, pullback pressure gradient index.

**

Supplemental Figure 3 The selection for tuning parameters

The top and bottom panels show the selection for tuning parameters in the two models used for classification on focal vs diffuse and focal vs non-focal, respectively. Each coloured line represents a different value of regularization parameter. The mixing percentage balances the lasso and ridge regression. The tuning parameters are selected based on highest accuracy.

μFR, Murray’s law-based quantitative flow ratio; PPGi, pullback pressure gradient index.

Supplemental Table 1 Three penalized logistic regression models

| Features | | | | | | |
| --- | --- | --- | --- | --- | --- | --- |
|  | Vessels (1 variable) | Demographics  (6 variables) | MFPCA  scores  (3 variables) | Indices | | |
|  |  |  |  | dμFR /ds | μFR | PPGi |
| μFR-model | √ | √ | √ | √ | √ |  |
| PPGi-model | √ | √ | √ | √ | √ | Quantitative |
