# Supplementary material for: Validation of machine learning angiography-derived physiological pattern of coronary artery disease
Source: Eur Heart J Digit Health. 2025 Apr 8;6(4):577–86. doi: 10.1093/ehjdh/ztaf031 (PMC12282386; doi:10.1093/ehjdh/ztaf031)
Supplement: ztaf031_Supplementary_Data [file ztaf031_supplementary_data.zip › Supplemental Fig3.pdf.pdf]

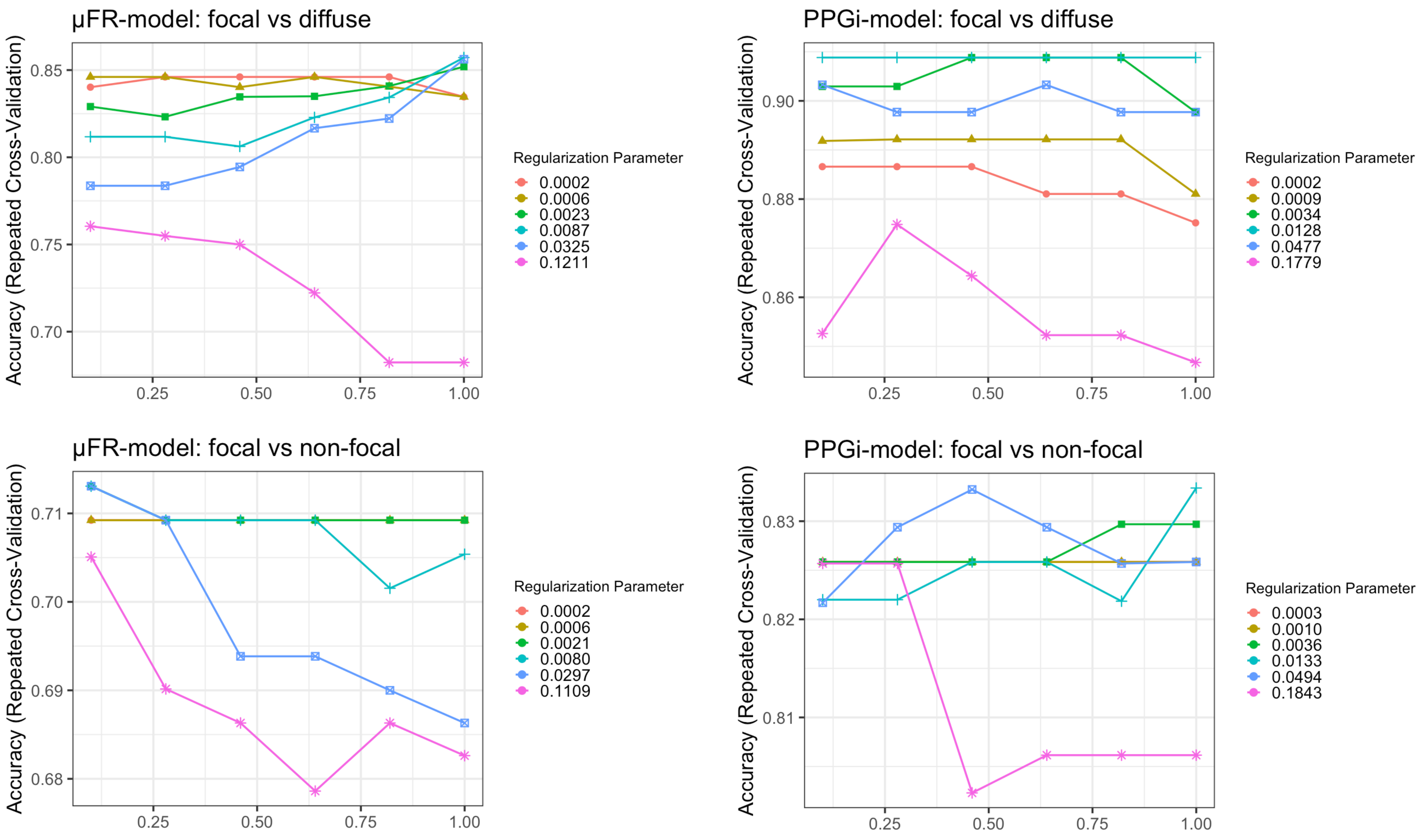
μFR-model: focal vs diffuse
Accuracy (Repeated Cross-Validation)
0.85
0.80
0.75
0.70
0.25
0.50
0.75
1.00
Regularization Parameter
0.0002
0.0006
0.0023
0.0087
0.0325
0.1211
PPGi-model: focal vs diffuse
Accuracy (Repeated Cross-Validation)
0.90
0.88
0.86
0.25
0.50
0.75
1.00
Regularization Parameter
0.0002
0.0009
0.0034
0.0128
0.0477
0.1779
μFR-model: focal vs non-focal
Accuracy (Repeated Cross-Validation)
0.71
0.70
0.69
0.68
0.25
0.50
0.75
1.00
Regularization Parameter
0.0002
0.0006
0.0021
0.0080
0.0297
0.1109
PPGi-model: focal vs non-focal
Accuracy (Repeated Cross-Validation)
0.83
0.82
0.81
0.25
0.50
0.75
1.00
Regularization Parameter
0.0003
0.0010
0.0036
0.0133
0.0494
0.1843
