# Supplementary figures and images for: Validation of machine learning angiography-derived physiological pattern of coronary artery disease
Source: Eur Heart J Digit Health. 2025 Apr 8;6(4):577–86. doi: 10.1093/ehjdh/ztaf031 (PMC12282386; doi:10.1093/ehjdh/ztaf031)

# MFPCA

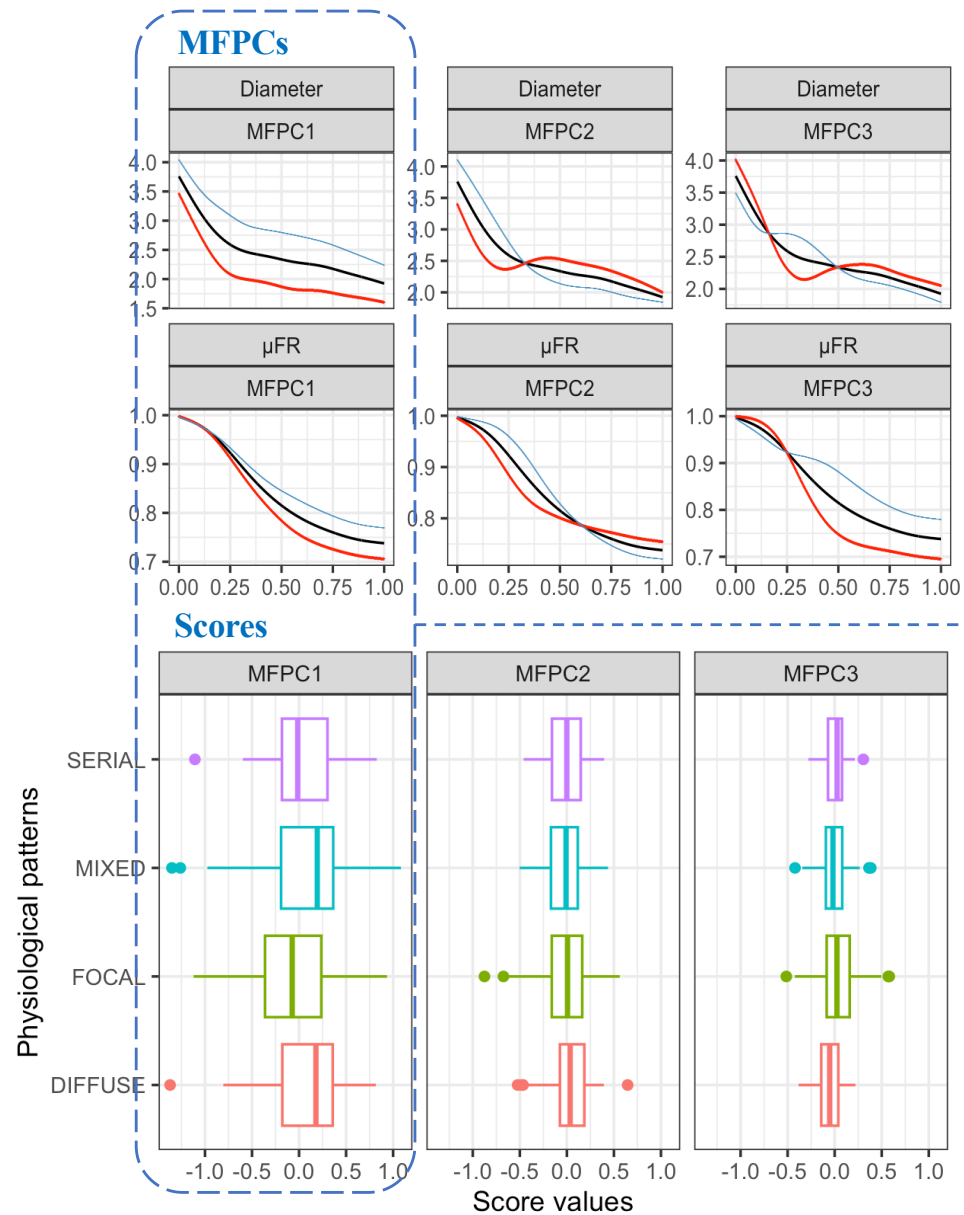

Curves (n=40) based on higher (red) and lower (blue) scores

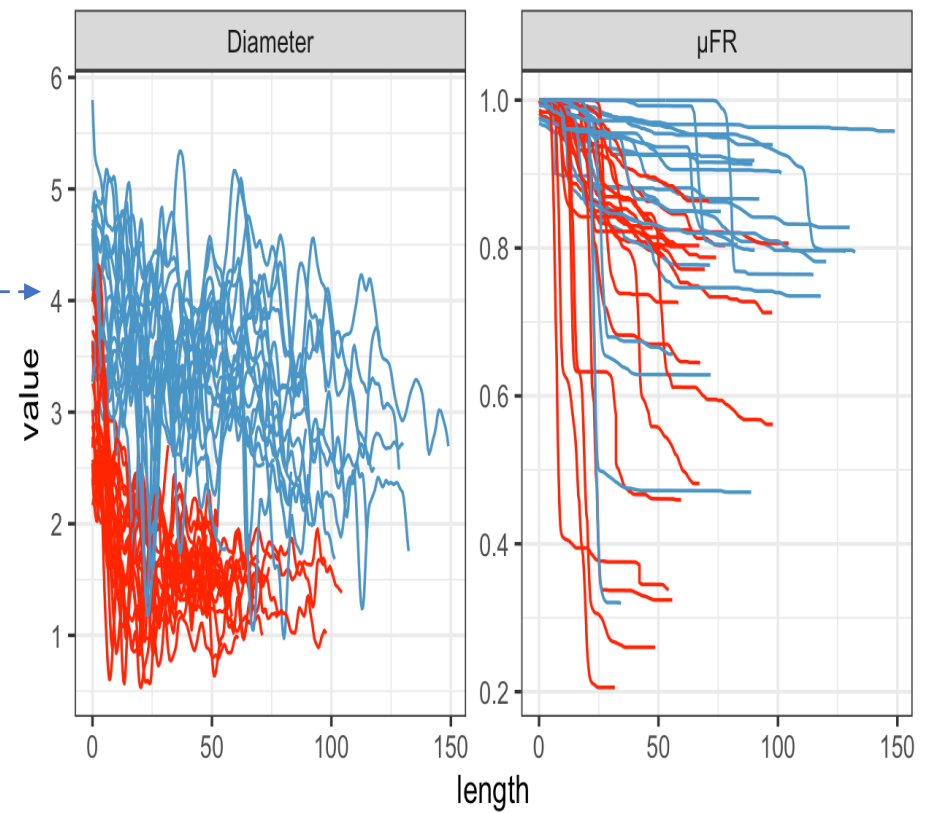

Supplement: ztaf031_Supplementary_Data [file ztaf031_supplementary_data.zip › Supplemental Fig1.pdf.pdf]

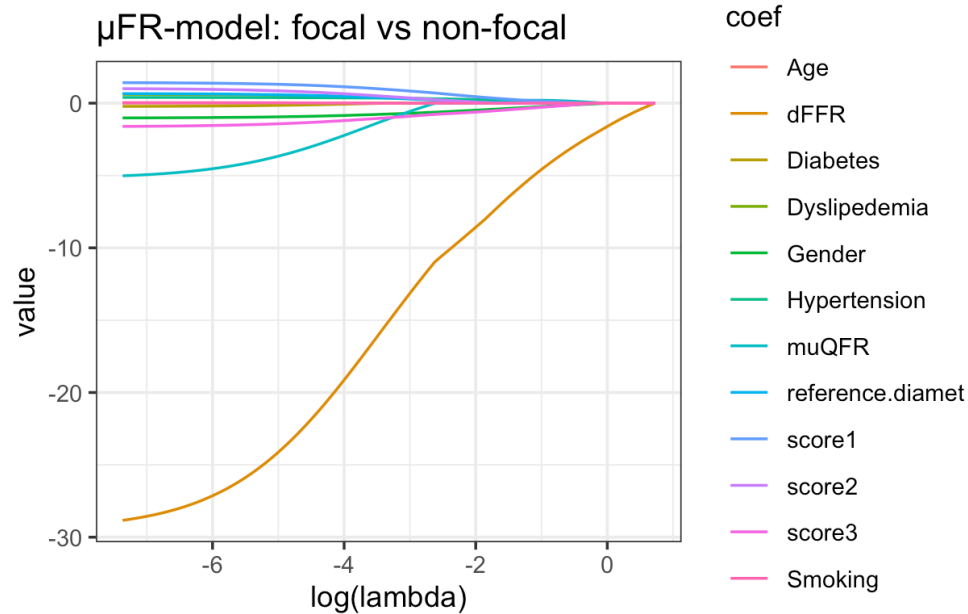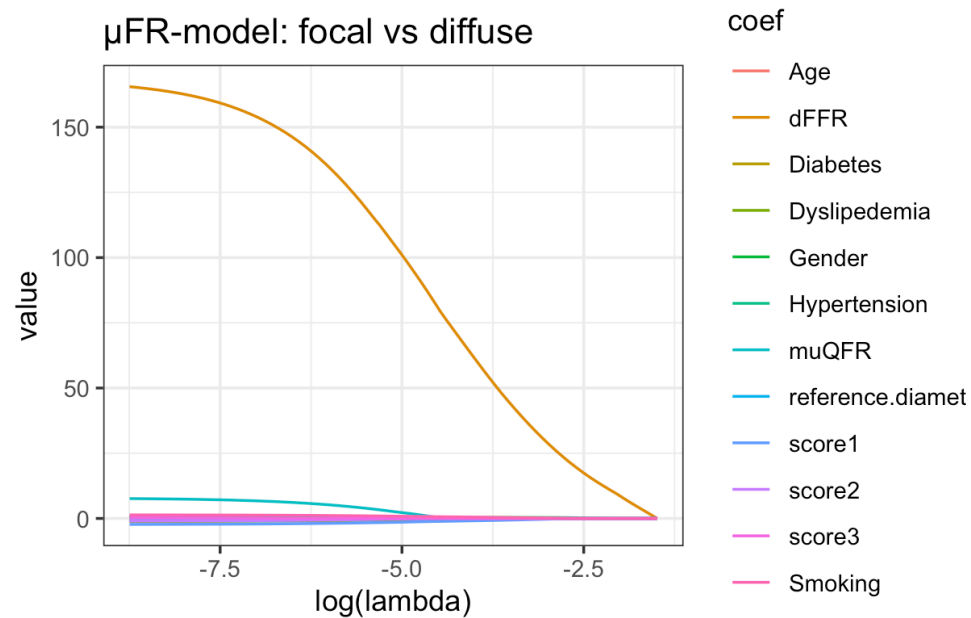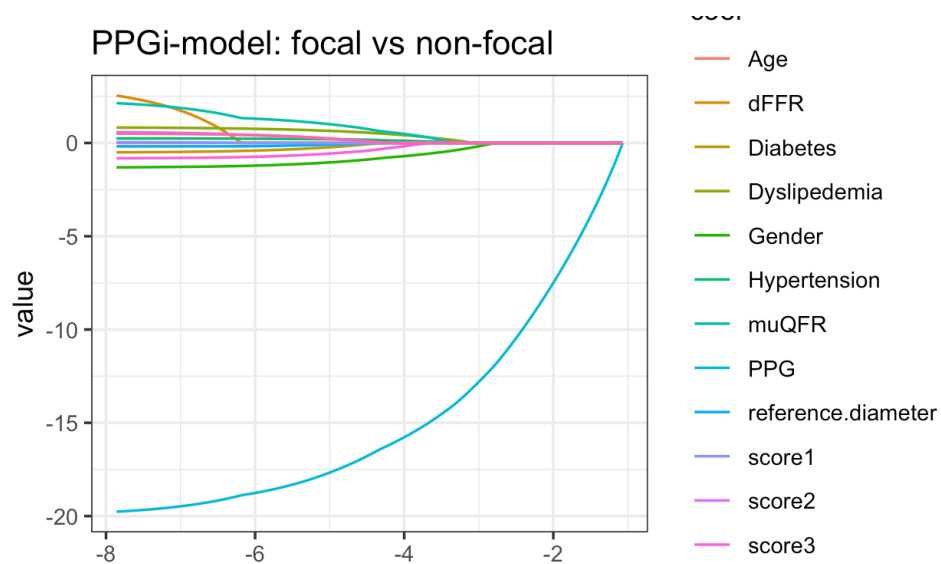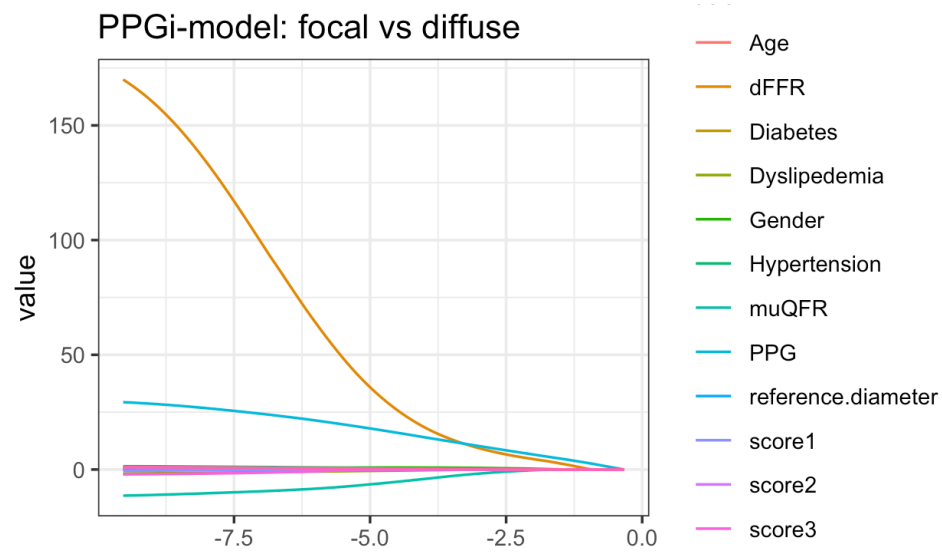

Supplement: ztaf031_Supplementary_Data [file ztaf031_supplementary_data.zip › Supplemental Fig2.pdf.pdf]
